# Nutrition Support in Liver Transplantation and Postoperative Recovery: The Effects of Vitamin D Level and Vitamin D Supplementation in Liver Transplantation

**DOI:** 10.3390/nu12123677

**Published:** 2020-11-28

**Authors:** Junshi Doi, Amika Moro, Masato Fujiki, Bijan Eghtesad, Cristiano Quintini, K. V. Narayanan Menon, Koji Hashimoto, Kazunari Sasaki

**Affiliations:** 1 Department of General Surgery, Digestive Disease & Surgery Institute, Cleveland Clinic, Cleveland, OH 44195, USA; doij@ccf.org (J.D.); moroa2@ccf.org (A.M.); fujikim@ccf.org (M.F.); eghtesb@ccf.org (B.E.); quintic@ccf.org (C.Q.); hashimk@ccf.org (K.H.); 2 Department of Gastroenterology and Hepatology, Cleveland Clinic, Cleveland, OH 44195, USA; menonk@ccf.org

**Keywords:** vitamin D, liver transplant, rejection, vitamin D supplementation, survival

## Abstract

Vitamin D plays an important role in the arena of liver transplantation. In addition to affecting skeletal health significantly, it also clinically exerts immune-modulatory properties. Vitamin D deficiency is one of the nutritional issues in the perioperative period of liver transplantation (LT). Although vitamin D deficiency is known to contribute to higher incidences of acute cellular rejection (ACR) and graft failure in other solid organ transplantation, such as kidneys and lungs, its role in LT is not well understood. The aim of this study was to investigate the clinical implication of vitamin D deficiency in LT. LT outcomes were reviewed in a retrospective cohort of 528 recipients during 2014–2019. In the pre-transplant period, 55% of patients were vitamin-D-deficient. The serum vitamin D level was correlated with the model for end-stage liver disease (MELD-Na) score. Vitamin D deficiency in the post-transplant period was associated with lower survival after LT, and the post-transplant supplementation of vitamin D was associated with a lower risk of ACR. The optimal vitamin D status and vitamin D supplementation in the post-transplant period may prolong survival and reduce ACR incidence.

## 1. Introduction

Vitamin D plays an important role in bone metabolism, regulating gene expression in multiple tissues, and increasing the intestinal absorption of calcium. Recently, in addition to the well-known effects on musculoskeletal metabolism, it has been reported that vitamin D has anti-inflammatory and immune-modulatory properties [1,2,3]. Clinically low serum levels of vitamin D have been associated with a higher prevalence of infections, cancer, cardiovascular, and autoimmune disorders [4,5]. Vitamin D deficiency is one of nutrition issues that is addressed in liver transplantation (LT) patients [6]. Due to the end-stage liver disease (ESLD) of the LT patients, malabsorption, inadequate dietary intake, and impairment in hepatic activation of vitamins are major issues [7,8]. While LT has been reported to have positive effects in increasing serum vitamin D concentrations as well as the percentage of patients with sufficient vitamin D levels, immunosuppression-related metabolic disturbances cause vitamin D deficiency after LT [9,10]. These malnutrition/vitamin deficiencies are challenges in LT management and can cause debility and worse postoperative outcomes [11].

Previous reports show that vitamin D has a protective effect against rejection and infection in the clinical settings of solid organ transplantation, such as kidney and lung. In addition, vitamin D supplementation is reported to lower the risk of acute cellular rejection (ACR) [12,13]. According to one of the previous reports, which reviewed a small number of patients, vitamin D supplementation in the post-transplant period was recommended for better outcomes after LT including decreased incidence of ACR and infections [14]. However, in terms of immunological aspects, the clinical effects of vitamin D on the outcome of LT have not been fully understood. Furthermore, the optimal method of vitamin D supplementation in clinical settings remains unclear.

In this study, we focused on vitamin D as a part of nutritional management in LT. We conducted a retrospective study (1) to evaluate the relationship between vitamin D deficiency and ESLD, (2) to analyze the relationships between serum levels of vitamin D and LT outcomes, and (3) to discuss the clinical effects of vitamin D supplementation.

## 2. Materials and Methods

### 2.1. Study Design and Population

This study included patients who underwent LT between 2014 and 2019 at Cleveland Clinic, Cleveland, Ohio. Patients who were under 18 years old, died during the perioperative period, underwent multiple organ transplantations or living donor liver transplant, developed primary non-function, or diagnosed with fulminant/acute liver failure for LT were excluded from the cohort.

Patient demographic and clinic-pathological data were extracted for the recipients including age, sex, body mass index (BMI), cause of cirrhosis, received hepatocellular carcinoma (HCC) exception, medical condition at the time of LT, history of previous abdominal surgery, portal vein thrombosis, model for end-stage liver disease (MELD-Na) score [15], and waiting time. For the donor, in addition to the basic characteristics, race, graft type, cause of death, share type, and cold ischemic time were extracted. Crossmatches of T and B cell results were also investigated. Crossmatching of donors against transplant recipients are performed to determine the likelihood of donor-specific antibody-mediated responses [16]. The primary end point was overall survival (OS) after LT and incidence of ACR. The presence of ACR was defined by biopsy. Follow-up was conducted for ACR incidence until patient death or 12 months after LT. This study was conducted in accordance with the Declaration of Helsinki, and the protocol was approved by the Institutional Review Board of Cleveland Clinic (IRB# 20-1169).

### 2.2. Post-Transplant Management

The immunosuppression protocol was based on tacrolimus with its initial target blood level at 8–10 ng/mL. Dosing was adjusted to whole blood concentrations and occurrence of renal impairment and other adverse effects. Immunosuppression was started immediately after LT, and oral medication was given once gut function was restored. In most cases, it was combined with tapering doses of corticosteroids and mycophenolate mofetil (2000 mg/day) [17]. 

### 2.3. Vitamin D Measurement

The serum levels of 25-hydroxy vitamin D (25(OH)D) were measured at the time of listing and at the post-transplant period of <1 year after LT as a part of routine nutrition evaluation. The serum level of 25(OH)D was considered low when <20 ng/mL [18].

### 2.4. Statistical Analysis

Continuous variables were reported as median with interquartile ranges (IQRs), and categorical variables were presented as frequencies (%). Continuous variables were compared using Student’s t-test or one/two-way ANOVA test, and categorical variables were compared using Fisher’s Exact tests or Chi-square test. Pearson’s rank correlation coefficient was used to test the association between calculated MELD-Na scores and serum levels of 25(OH)D. Kaplan–Meier curves were constructed and compared using a log-rank test. To evaluate the factors associated with survival after LT, Cox regression analysis was performed. Cumulative incidences of ACR were calculated using the Fine–Gray competing risk approach [19]. To evaluate the risk factors associated with ACR, Fine–Gray proportional hazards regression analysis was performed. Hazard ratio (HR) and subdistribution hazard ratio (sHR) are presented with 95% confidence intervals (95% CI). A *p*-value < 0.05 was considered statistically significant. All statistical analyses were performed with EZR (Saitama Medical Center, Jichi Medical University, Saitama, Japan), which is a graphical user interface for R (The R Foundation for Statistical Computing, Vienna, Austria) [20]. 

## 3. Results

### 3.1. Demographical Characteristics of Patients

**Figure 1:**
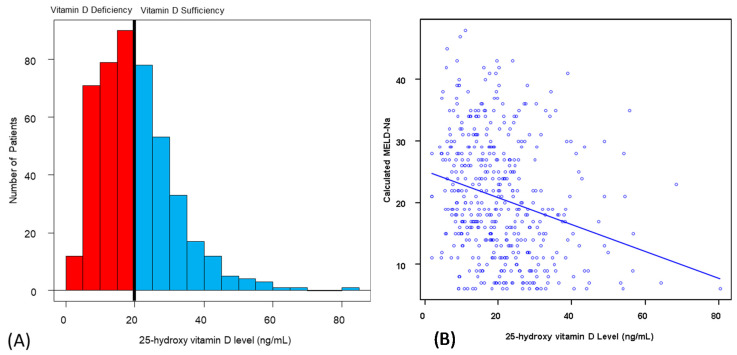
25-hydroxy vitamin D levels in patients prior to LT. (**A**) Distribution of the 25-hydroxy vitamin D levels in the pre-transplant period. (**B**) Correlation between the 25-hydroxy vitamin D levels and MELD-Na score. Correlation coefficient r was −0.254 (*p* < 0.01; 95% CI: −0.34–−0.17). CI: confidence interval; MELD-Na: the model for end-stage liver disease. LT: liver transplantation.

A total of 528 patients were included in the analytic cohort (Table 1). The median recipient age at the time of LT was 58 years (IQR: 52–64), and more than half of the recipients were male (*n* = 350, 66.2%). Cause of cirrhosis was mainly alcohol (*n* = 154, 29.1%), NASH/Cryptogenic (*n* = 136, 25.9%), and viral hepatitis (*n* = 103, 19.5%). Half of the recipients received previous abdominal surgery (*n* = 266, 50.3%), and a minority of recipients had portal vein thrombosis at the time of LT (*n* = 104, 19.7%). The median laboratory MELD-Na score was 19 (IQR: 13–28). The median waiting time was 2.9 months (IQR: 0.8–7.4). The majority of the donor graft type was donor after brain dead (DBD) (*n* = 458, 86.8%). The median cold ischemic time was 6.2 h (IQR: 5.3–7.4). Figure 1A shows the distribution of vitamin D status prior to LT, showing 55% were vitamin-D-deficient. The characteristics of vitamin-D-deficient and -sufficient patients were compared (Table 2). Recipient characteristics including age >60 years, presence of HCC, alcohol consumption rate, MELD-Na score, and serum albumin level had a significant difference (*p* < 0.05). The relationship between the MELD-Na score and serum levels of vitamin D in the pre-transplant period was analyzed (Figure 1B). The correlation coefficient was −0.254 (*p* < 0.01; 95% CI: −0.34–−0.17).

### 3.2. Influence of Preoperative and Postoperative Serum Vitamin D Levels on Overall Survival 

**Figure 2:**
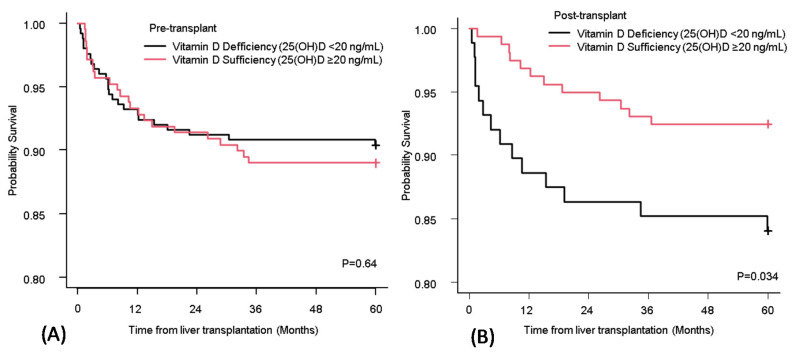
OS of recipients who had vitamin D deficiency and sufficiency at pre-transplant (**A**) and post-transplant (**B**). The Kaplan–Meier curve for OS of recipients who had vitamin D deficiency (black line) and sufficiency (red line). Vitamin D deficiency was defined as <20 ng/mL of the serum level of 25(OH)D. OS: overall survival; 25(OH)D: 25-hydroxy vitamin D.

Differences in the long-term survival between patients who had vitamin D deficiency and sufficiency at pre- and post-transplant were compared (Figure 2A,B). There was no significant difference between the patients who had vitamin D deficiency and sufficiency in the pre-transplant period (*p* = 0.64) (Figure 2A). However, there was a significant difference between the two groups in the post-transplant period (Figure 2B). 

A bivariate and multivariable Cox regression analysis was performed to assess the risk factors associated with the five-year OS (Table 3). Older age (>60 years old) (HR 3.47; 95% CI, 1.38–8.68, *p* < 0.01) and post-transplant vitamin D sufficiency (HR 0.31; 95% CI, 0.13–0.75, *p* < 0.01) were associated with five-year OS.

### 3.3. Influence of Preoperative and Postoperative Serum Vitamin D Levels on Acute Cellular Rejection

**Figure 3:**
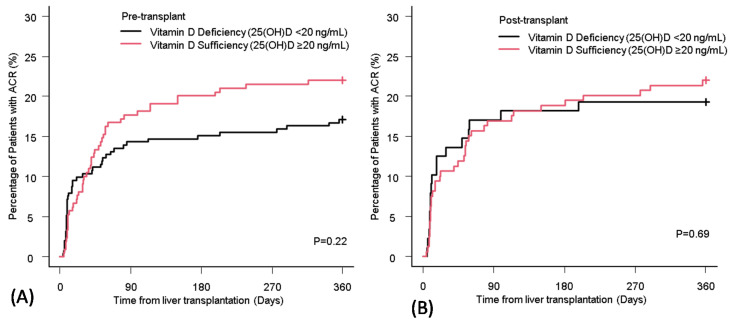
The cumulative incidence of ACR in liver transplant recipients with vitamin D deficiency and sufficiency at pre-transplant (**A**) and post-transplant (**B**). ACR: acute cellular rejection; 25(OH)D: 25-hydroxy vitamin D.

The incidence of ACR in LT recipients with pre- and post-transplant vitamin D deficiency was 19.1% and 22.0%, respectively. There was no significant difference in the cumulative incidence of ACR between vitamin D deficiency and sufficiency in both pre- and post-transplant periods (Figure 3A,B).

### 3.4. Comparison of Patient Characteristics Based on Vitamin D Supplementation Status

In examining the effect of vitamin D supplementation, four groups were investigated: (1) patients who did not receive supplementation (No Supplement), (2) patients who received supplementation during only pre-transplant (Pre), (3) patients who received supplementation during only post-transplant (Post), and (4) patients who received supplementation during both pre- and post- transplant (Pre/Post). The characteristics of the four groups were compared (Table 4). Among the four groups, there was a significant difference in the ratio of sex and 25(OH)D level at pre-transplant (*p* < 0.05).

### 3.5. Effect of Vitamin D Supplementation on Overall Survival

**Figure 4:**
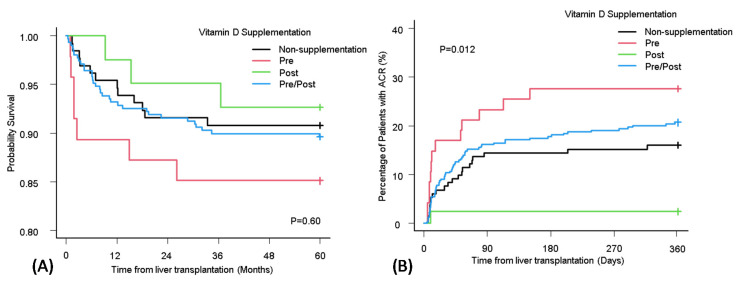
Comparison of vitamin D supplement status in OS (**A**) and the accumulate incidence of ACR (**B**). ACR: acute cellular rejection; OS: overall survival.

Differences in the long-term survival among the four patient groups (No Supplement, Pre, Post, Pre/Post) were compared using the Kaplan–Meier curve (Figure 4A). Regardless of the supplementation status, there were no significant differences in the OS (*p* = 0.60).

### 3.6. Effect of Vitamin D Supplementation on Acute Cellular Rejection

The cumulative incidence of ACR among the four groups (No Supplement, Pre, Post, Pre/Post) (Figure 4B) was compared. Interestingly, the incidence rate of ACR showed a significant difference based on the vitamin D supplementation status. The cumulative incidence was high in the Pre group and was low in the Post group. 

The proportional subdistribution hazard model of the Fine and Gray method was used for ACR in the No Supplement group and Pre group (Table 5). From the bivariate and multivariable analysis, age (>60 years) was the only variable that was significant (sHR 0.30; 95% CI, 0.12–0.77, *p* = 0.01). In the same manner, the Fine and Gray method was used for ACR in the No Supplement group and Post group (Table 6). Among all variables, calculated MELD-Na score >30 (sHR < 0.01; 95% CI, <0.01–<0.01, *p* < 0.01) at the time of LT and vitamin D supplementation during post-transplant (sHR 0.09; 95% CI, 0.01–0.72, *p* = 0.02) were significant.

## 4. Discussion

Although previous studies have reported that vitamin D plays an important role in solid organ transplantation including kidney and lung [12,13], the clinical impact of vitamin D on LT outcomes and vitamin D supplementation is still unknown. The current study included 528 patients which is larger than any other previous studies of vitamin D in LT. Our study investigated the correlation of the pre-transplant vitamin D level and the MELD-Na score. Moreover, the current study is important because we were able to reveal how the perioperative vitamin D levels and vitamin D supplementation status affect long-term outcomes, such as OS and ACR all in the same cohort. Using the cutoff of 20 ng/mL of 25(OH)D [18], 55% of the patients had vitamin D deficiency before LT. The MELD-Na score and serum levels of 25(OH)D before LT showed a negative linear relationship. While there was no survival difference based on the vitamin D level during pre-transplant, patients who had vitamin D deficiency at post-transplantation had worse survival compared with vitamin-D-sufficient patients. The cumulative incidence of ACR was not affected by the perioperative 25(OH)D level. There was no difference in the OS when it was assessed based on the vitamin D supplementation status. However, the accumulated incidence of ACR was high in the Pre group and low in the Post group, showing a significant difference. Importantly, the risk factor of ACR in the Pre group and Post group was younger age and no vitamin D supplementation and higher MELD-Na score at the time of LT, respectively. 

Vitamin D3 is taken in the body by diet (20%) or is synthesized by the skin (80%) from 7-dihydrocholesterol following UVB exposure. Vitamin D3 becomes biologically active after hydroxylation in the liver by the enzymes cytochrome P450 2R1 and cytochrome P450 27 becoming 25-hydroxyvitamin D3. The fully active metabolite 1,25-dihydroxyvitamin D3 is hydroxylated in the kidney [21]. ESLD patients have both impaired liver and kidney function that can alter calcium and vitamin D homeostasis [22,23]. Even when there were patients taking supplemental vitamin D during pre-transplant, more than half of the patients had vitamin D deficiency (Figure 1A). In addition, we found that there was a negative correlation between MELD-Na scores and serum levels of 25(OH)D in the pre-transplant period, which was compatible with the vitamin D physiology since the MELD-Na score incudes both hepatic and renal components [15].

Vitamin-D-sufficient status in the post-transplant period was associated with five-year survival after LT. The optimal vitamin D status prolonged survival. This indicated that post-transplant nutritional support including the correction of vitamin D deficiency will support better OS. These results are consistent with a study from Lowery et al., showing that the mortality of lung transplant recipients who remained vitamin-D-deficient at one-year post-transplant was higher than that of recipients who maintained normal vitamin D levels [13]. In the current study, the six-month mortality rate of vitamin-D-deficient patients in the post-transplant period was 8.0% while that in vitamin-D-sufficient patients was 0.63% (*p* < 0.01) (Figure 2B). Post-transplant vitamin-D-deficient status had larger effects on mortality in the early period after LT compared with the late period. Even though early mortality after LT can be caused by different conditions such as early allograft dysfunction and infections [24], these complications might be related to decreased immune-modulatory properties because of the low vitamin D level [1,2,3]. On the other hand, low vitamin D can be a result of the malnourished condition of LT recipients. Malnutrition itself could also negatively affect mortality [25,26]. Thus, serum levels of 25(OH)D in the post-transplant period can be a prognostic factor or a predictive factor for survival. Further investigation to determine how the vitamin D status contributes to OS is needed.

Pre-transplant vitamin D status was not associated with the long-term accumulated incidence of ACR after LT in this study (Figure 3A). Although focusing on the short term after LT, the vitamin D deficiency group had a higher rate of ACR compared to the sufficiency group. Similar findings were seen in a paper from Zhou et al. showing that high pre-transplant 25(OH)D level (>25 ng/mL) prior to LT significantly decreased the incidence of ACR in 30 days after LT [27]. Another report from Bitetto et al. also confirmed that low pre-transplant 25(OH)D level (<5 ng/mL) was independently associated with moderate to severe ACR episodes within two months after LT [28]. After the early period of LT, the incidence of ACR in the vitamin D sufficiency group tended to be higher than the deficiency group. This implies that the vitamin D sufficiency in the pre-transplant period contributed to reduced risks for ACR in the early post-transplant period and smoothly boosted the immune system of recipients through the recovery phase from LT-related surgical procedures in the late post-transplant period. The vitamin D status during the post-transplant period had no significant relationship with the incidence of ACR (Figure 3B).

As such, pre-transplant vitamin D levels may be associated with ACR in the early period after LT, and it can be hypothesized that optimization of vitamin-D-deficient status by supplementation in the pre-transplant period may contribute to reducing the incidence of ACR.

The clinical effects of vitamin D supplementation on LT outcomes remained unclear. We demonstrated that vitamin D supplementation in the post-transplant period has positive effects in decreasing the incidence of ACR during one year after LT. A previous study showed similar findings that vitamin D supplementation for the first one month significantly decreased the incidence of ACR in 30 days after LT [27]. Vitamin D supplementation is assumed to increase the components of suppressor T cells/T memory cells, decrease the C3 co-stimulatory molecule expression (HLA-DR, CD28), and expand T naïve cells/cytotoxic T cells [27,29]. Our comparison between the No Supplement and the Post groups clarified the anti-rejection effects of the post-transplant vitamin D supplementation. From these results, we propose that vitamin D supplementation should be considered, especially to reduce the incidence of ACR. Yet, our result does not demonstrate that vitamin D supplementation in the pre-transplant period could reduce the incidence of ACR. Thus, prospective studies should be conducted to elucidate the importance of vitamin D supplementation in LT, including the relationship between vitamin D supplementation and modification of vitamin D levels.

This study had several limitations. Since this was a retrospective study, information bias was possible given that the data were manually abstracted from the medical records. Additionally, there was potential for unmeasured confounders of the relationship between vitamin D status and hepatic graft status. The multivariable analysis of all four groups (No Supplement, Pre, Post, Pre/Post) showed that sex is not a significant factor regulating the incidence of ACR (Table 5 and Table 6) or five-year survival (data not shown), although there were gender differences among groups (Table 4). In this study, more female patients tended to be on vitamin D supplementation compared with male patients. We assume it was because of the higher incidence of bone-related disorders in female groups, such as osteoporosis. Future research is needed to elucidate the gender effects on outcomes after LT.

Finally, it should be noted that there are no guidelines for screening or supplementation of vitamin D in LT recipients. Further investigation is needed to clarify the importance of screening for vitamin D status and the effectiveness of vitamin D supplementation for patients in the peri-transplant period.

## 5. Conclusions

Vitamin D deficiency in the post-transplant period was associated with lower survival after LT, and the post-transplant supplementation of vitamin D was associated with a lower risk of ACR. Vitamin D levels in the pre-transplant period may be an important factor of ACR. Nutritional support with vitamin D supplementation might be contributing to improving LT outcomes.

## Figures and Tables

**Table 1:** Recipient and donor characteristics. LT: liver transplantation.

**Recipient Characteristics**	
Age, years, Median (IQR)	58 (52–64)
Sex, male, Number (%)	350 (66.2)
Height, cm, Median (IQR)	174.0 (165.8–180.3)
Weight, kg, Median (IQR)	86.2 (72.3–101.3)
Body mass index, kg/m^2^, Median (IQR)	28.7 (24.8–33.8)
Cause of cirrhosis, Number (%)	
Viral hepatitis	103 (19.5)
PBC/PSC/AIH	85 (16.1)
NASH/Cryptogenic	136 (25.9)
Alcohol	154 (29.1)
Other	50 (9.5)
Exception for HCC, Number (%)	202 (17.7)
Medical condition at the time of LT, Number (%)	
ICU	39 (7.4)
Hospitalized	207 (39.5)
Home	280 (53.1)
Previous abdominal surgery, Number (%)	266 (50.3)
Portal vein thrombosis, Number (%)	104 (19.7)
MELD-Na score, Median (IQR)	19 (13–28)
Waiting time, months, Median (IQR)	2.9 (0.8–7.4)
**Donor Characteristics**	
Age, years, Median (IQR)	42 (30–54)
Sex, male, Number (%)	317 (59.9)
Height, cm, Median (IQR)	173.0 (165.0–180.0)
Weight, kg, Median (IQR)	82.1 (69.9–99.4)
Body mass index, kg/m^2^, Median (IQR)	28.1 (23.68–32.9)
Graft type, Number (%)	
DBD	429 (81.3)
DCD	65 (12.3)
Split	34 (6.4)
Race/Ethnicity, Number (%)	
White	410 (77.7)
African American	93 (17.6)
Others	25 (4.7)
Cause of death, Number (%)	
Head trauma	251 (47.6)
Anoxia	150 (28.4)
CVD	120 (22.7)
Others	7 (1.3)
Share type, Number (%)	
Local	300 (56.9)
Regional	180 (34.0)
National	48 (9.1)
Cold ischemic time, hours, Median (IQR)	6.2 (5.3–7.4)

IQR: interquartile range; PBC: primary biliary cirrhosis; PSC: primary sclerosing cholangitis; AIH: autoimmune hepatitis; NASH: non-alcoholic steatohepatitis; HCC: hepatocellular carcinoma; ICU: intensive care unit; MELD-Na: model for end-stage liver disease; DBD: donor after brain dead; DCD: donor after circulation death; CVD: cardiovascular disease.

**Table 2:** Characteristics of vitamin-D-deficient and -sufficient patients prior to liver transplantation.

	All Patients		
	Deficient (20 ng/mL<)	Sufficient (≥20 ng/mL)	*p* Value
Characteristics			
Age >60 years, Number (%)	93 (37.1)	108 (51.7)	<0.01
Sex, Number (%)			0.37
Male	173 (68.9)	135 (64.6)	
Female	78 (31.1)	74 (35.4)	
Body mass index, kg/m^2^, Median (IQR)	28.7 (24.7–34.1)	28.6 (25.0–33.3)	0.90
Main liver disease, Number (%)			0.08
HBV/HCV	37 (14.7)	49 (23.4)	
PBC/PSC/AIH	40 (15.9)	34 (16.3)	
NASH	68 (27.1)	54 (25.8)	
Alcohol	83 (33.1)	50 (23.9)	
Other	23 (9.2)	22 (10.5)	
Hepatocellular carcinoma, Number (%)	64 (25.5)	77 (36.8)	0.01
Diabetes mellitus, Number (%)	70 (27.9)	66 (31.6)	0.41
Alcohol consumption, Number (%)	83 (33.1)	50 (23.9)	0.04
MELD-Na score, Median (IQR)	22 (16–29)	16 (10–24)	<0.01
Albumin, g/dL, Median (IQR)	3.0 (2.6–3.5)	3.2 (2.8–3.7)	0.04
25(OH)D, Pre-transplant, ng/mL, Median (IQR)	12.9 (9.1–16.5)	27.0 (23.1–32.9)	<0.01
25(OH)D, Post-transplant, ng/mL, Median (IQR)	22.1 (14.4–29.0)	27.1 (20.3–35.5)	0.32
Crossmatch for T and B cell, Number (%)			0.24
T cell: −/B cell: −	154 (61.4)	129 (61.7)	
T cell: +/B cell: −	16 (6.4)	9 (4.3)	
T cell: −/B cell: +	47 (18.7)	51 (24.4)	
T cell: +/B cell: +	34 (13.5)	20 (9.6)	

IQR: interquartile range; HBV: hepatitis B virus; HCV: hepatitis C virus; PBC: primary biliary cirrhosis; PSC: primary sclerosing cholangitis; AIH: autoimmune hepatitis; NASH: non-alcoholic steatohepatitis; MELD-Na: model for end-stage liver disease; 25(OH)D: 25-hydroxy vitamin D.

**Table 3:** Cox regression analysis of prognostic factors associated with overall survival.

	Bivariate Analysis		Multivariable Analysis	
Variable	HR (95% CI)	*p*-Value	HR (95% CI)	*p* Value
Age >60 years	2.84 (1.60–5.05)	<0.01	3.47 (1.38–8.68)	<0.01
Sex (Male)	0.79 (0.46–1.36)	0.39	0.65 (0.28–1.54)	0.33
Body mass index	0.87 (0.50–1.50)	0.61		
MELD-Na score >30	1.70 (0.94–3.04)	0.08	1.02 (0.97–1.06)	0.47
Hepatocellular carcinoma	1.48 (0.85–2.55)	0.16		
Alcohol-related cirrhosis	0.75 (0.47–1.19)	0.22		
Vitamin D sufficiency, Pre-transplant	1.15 (0.65–2.03)	0.63	1.02 (0.42–2.44)	0.97
Vitamin D sufficiency, Post-transplant	0.44 (0.21–0.96)	0.04	0.31 (0.13–0.75)	<0.01
Vitamin D supplementation, Pre-transplant	1.28 (0.71–2.32)	0.41		
Vitamin D supplementation, Post-transplant	0.92 (0.53–1.61)	0.78		
Crossmatch for T or B cell positive	1.26 (0.85–1.87)	0.26		

CI: confidence interval; HR: hazard ratio; MELD-Na: model for end-stage liver disease.

**Table 4:** Comparison of patient characteristics among different vitamin D supplementation statuses.

	All Patients (*N* = 528)				
Characteristics	No Supplementation *N* = 131	Pre *N* = 47	Post *N* = 41	Pre/Post *N* = 309	*p* Value
Age >60 years, Number (%)	65 (49.6)	18 (38.3)	14 (34.1)	138 (44.7)	0.27
Sex, Number (%)					0.03
Male	98 (74.8)	29 (61.7)	31 (75.6)	191 (61.8)	
Female	33 (25.2)	18 (38.3)	10 (24.4)	118 (38.2)	
Body mass index, kg/m^2^, Median (IQR)	28.7 (24.7–33.8)	28.5 (22.7–33.1)	29.0 (25.5–33.3)	28.7 (24.9–34.0)	0.98
Main liver disease, Number (%)					0.12
HBV/HCV	35 (26.7)	11 (23.4)	5 (12.2)	52 (16.8)	
PBC/PSC/AIH	15 (11.5)	8 (17.0)	3 (7.3)	59 (19.1)	
NASH	32 (24.4)	11 (23.4)	14 (34.1)	80 (25.9)	
Alcohol	38 (29.0)	14 (29.8)	11 (26.8)	90 (29.1)	
Other	11 (8.4)	3 (6.4)	8 (19.5)	28 (9.1)	
Hepatocellular carcinoma, Number (%)	44 (33.6)	15 (31.9)	11 (26.8)	88 (28.5)	0.70
Diabetes mellitus, Number (%)	40 (30.5)	17 (36.2)	9 (22.0)	87 (28.2)	0.49
Alcohol consumption, Number (%)	38 (29.0)	14 (29.8)	11 (26.8)	90 (29.1)	0.99
MELD-Na score, Median (IQR)	19 (13–28)	18 (11–27)	22 (15–28)	19 (14–28)	0.82
Albumin, g/dL, Median (IQR)	3.0 (2.6–3.5)	3.1 (2.8–3.5)	3.10 (2.4–3.8)	3.2 (2.7–3.6)	0.88
25(OH)D, Pre-transplant, ng/mL, Median (IQR)	18.1 (11.9–24.4)	14.3 (9.2–22.6)	17.65 (10.5–21.5)	20.2 (13.8–28.4)	<0.01
25(OH)D, Post-transplant, ng/mL, Median (IQR)	23.6 (19.4–33.6)	23.2 (13.1–29.3)	18.9 (11.4–23.9)	25.5 (18.2–34.1)	0.36
Crossmatch for T and B cell, Number (%)					0.12
T cell: −/B cell: −	83 (63.4)	26 (55.3)	32 (78.0)	180 (58.3)	
T cell: +/B cell: −	7 (5.3)	2 (4.3)	2 (4.9)	19 (6.1)	
T cell: −/B cell: +	30 (22.9)	15 (31.9)	7 (17.1)	66 (21.4)	
T cell: +/B cell: +	11 (8.4)	4 (8.5)	0 (0.0)	44 (14.2)	

IQR: interquartile range; HBV: hepatitis B virus; HCV: hepatitis C virus; PBC: primary biliary cirrhosis; PSC: primary sclerosing cholangitis; AIH: autoimmune hepatitis; NASH: non-alcoholic steatohepatitis; MELD-Na: model for end-stage liver disease; 25(OH)D: 25-hydroxy vitamin D.

**Table 5:** Fine and Gray proportional subdistribution hazard model for ACR development in No Supplement and Pre group.

	Bivariate Analysis		Multivariable Analysis	
Variables	sHR (95% CI)	*p* Value	sHR (95% CI)	*p* Value
Age >60 years	1.03 (0.42–2.53)	0.95	0.30 (0.12–0.77)	0.01
Sex (Male)	1.02 (0.50–2.08)	0.95		
Calculated MELD-Na >30	0.34 (0.08–1.43)	0.14	0.28 (0.07–1.17)	0.08
Hepatocellular carcinoma	0.95 (0.47–1.94)	0.89		
Alcohol-related cirrhosis	1.00 (0.48–2.06)	0.99		
Vitamin D supplementation, Pre-transplant	1.39 (0.67–2.87)	0.38	1.05 (0.43–2.57)	0.92
Vitamin D sufficiency, Pre-transplant	1.23 (0.59–2.59)	0.58	1.38 (0.63–3.01)	0.42
Crossmatch for T or B cell positive	0.94 (0.48–1.86)	0.86		

CI: confidence interval; sHR: subdistribution hazard ratio; MELD-Na: model for end-stage liver disease.

**Table 6:** Fine and Gray proportional subdistribution hazard model for ACR development in No Supplement and Post group.

	Bivariate Analysis		Multivariable Analysis	
Variables	sHR (95% CI)	*p* Value	sHR (95% CI)	*p* Value
Age >60 years	0.42 (0.17–1.06)	0.07	0.38 (0.07–2.02)	0.26
Sex (Male)	0.91 (0.36–2.27)	0.84		
Calculated MELD-Na > 30	0.34 (0.08–1.43)	0.14	<0.01 (<0.01–<0.01)	<0.01
Hepatocellular carcinoma	1.24 (0.52–2.93)	0.63		
Alcohol-related cirrhosis	1.44 (0.61–3.40)	0.40		
Vitamin D supplementation, Post-transplant	0.14 (0.02–1.07)	0.06	0.09 (0.01–0.72)	0.02
Vitamin D sufficiency, Post-transplant	0.85 (0.18–4.04)	0.84	0.69 (0.14–3.48)	0.65
Crossmatch for T or B cell positive	1.71 (0.74–3.91)	0.21		

CI: confidence interval; sHR: subdistribution hazard ratio; MELD-Na: model for end-stage liver disease.
